# The clinical efficacy of “water-jet” hemostasis in gastrointestinal endoscopic submucosal dissection

**DOI:** 10.1093/gastro/goae088

**Published:** 2024-09-26

**Authors:** Ran Chen, Qingyong Zhang, Shiya Hong, Fengying Chen, Xiaoqing Huang, Xiongfei Bao, Zhi Ni, Rongchun Zhang

**Affiliations:** Department of Gastroenterology, Xiamen Humanity Hospital, Fujian Medical University, Xiamen, Fujian, P. R. China; Department of Gastroenterology, Xiamen Humanity Hospital, Fujian Medical University, Xiamen, Fujian, P. R. China; Department of Gastroenterology, Xiamen Humanity Hospital, Fujian Medical University, Xiamen, Fujian, P. R. China; Department of Gastroenterology, Xiamen Humanity Hospital, Fujian Medical University, Xiamen, Fujian, P. R. China; Department of Gastroenterology, Xiamen Humanity Hospital, Fujian Medical University, Xiamen, Fujian, P. R. China; Department of Gastroenterology, Xiamen Humanity Hospital, Fujian Medical University, Xiamen, Fujian, P. R. China; Department of Gastroenterology, Xiamen Humanity Hospital, Fujian Medical University, Xiamen, Fujian, P. R. China; Department of Gastroenterology, Xiamen Humanity Hospital, Fujian Medical University, Xiamen, Fujian, P. R. China

**Keywords:** “water-jet”, endoscopic submucosal dissection, hemostasis, bleeding

## Abstract

**Objective:**

This study aims to evaluate the safety and efficacy of “water-jet” hemostasis during endoscopic submucosal dissection.

**Methods:**

In this prospective single-arm clinical study, 10 patients aged 18–60 years with gastric or intestinal mucosal lesions who were admitted to Fujian Medical University Xiamen Humanity Hospital (Xiamen, P. R. China) between June 2022 and June 2023 and met the absolute indications for endoscopic treatment were finally analyzed. The primary outcomes of this study are the incidence rates of adverse events and R0 resection, and the secondary outcomes are length of hospital stay and short- and long-term outcomes.

**Results:**

Successful hemostasis was achieved in all the included cases. In one case, the “water-jet” hemostasis failed to stop bleeding in one blood vessel, so the hemostatic forceps were used instead. No adverse events occurred in all cases. Pathologic results showed R0 resection in all samples.

**Conclusion:**

The “water-jet” method is safe and feasible for hemostasis in endoscopic submucosal dissection.

## Introduction

Digestive endoscopy plays an increasingly important role in the early diagnosis and treatment of gastrointestinal cancers. In particular, endoscopic submucosal dissection (ESD) can remove the early cancer and precancerous lesions at once and achieve a complete resection effect comparable to surgery. Bleeding during mucosal dissection is a crucial factor that has a significant impact on surgical procedures and is a potential threat to patient safety. Effectively and safely controlling bleeding is the fundamental requirement for successful endoscopic treatment [1, 2].

Intraoperative hemostasis in ESD can be divided into two parts: (1) pretreatment of free blood vessels for hemostasis during operation: submucosal blood vessels were exposed during submucosal injection and dissection of the lesion; the surgeon dissociates the blood vessels and exposes them for pretreatment to prevent bleeding; (2) intraoperative active bleeding hemostasis method: active bleeding occurred because undiscovered or unpretreated blood vessels were cut off during the process of stripping the lesion, and the bleeding point was stopped by the surgeon. If the blood vessels are not treated well during the process of hemostasis, it will have adverse effects on the follow-up operation. On the one hand, it is likely to rebleed during the operation, and the surgical field is not good and the surgical level is not clear, which not only affects the lesion dissection, but also easily damages the specimen and affects the pathological diagnosis. On the other hand, failure to properly handle the broken vessels can easily cause delayed postoperative bleeding, which is harmful to patients. Therefore, we explored the commonly used hemostasis methods in clinical practice. We found that when the water injection knife is used for surgical treatment, the strong coagulation and hemostasis at the edge of the water injection knife can safely and effectively pretreat the blood vessels. It can not only be used to pretreat blood vessels above 1 mm in diameter to stanch bleeding, reduce the use of thermocoagulation forceps, and reduce the time consumed by accessory exchange, but also help to quickly find the bleeding point, shorten the hemostasis time, and avoid the formation of eschar during hemostasis. We named it the “water-jet” hemostasis method. The purpose of this study was to investigate the safety and efficacy of this hemostatic method.

## Methods

### Patients

This study was a prospective single-arm clinical study. Patients aged 18–60 years with gastric or intestinal mucosal lesions who were admitted to Fujian Medical University Xiamen Humanity Hospital (Xiamen, P. R. China) between June 2022 and June 2023 and met the absolute indications for endoscopic treatment [3] were included.

Exclusion criteria were as follows: (i) patients with severe cardiopulmonary disease, coagulopathy, or mental illness; (ii) long-term use of anticoagulant or antiplatelet drugs; (iii) pregnant women; and (iv) intraoperative perforation not caused by “water-jet” hemostasis.

The “water-jet” technique was used to control intraoperative bleeding and manage postoperative wound care. All wounds were not sutured after operation.

Written informed consent was obtained from patients before surgery. This study complied with the relevant ethical requirements of the Declaration of Helsinki (Ethics No.: HAXM-MEC-20240130-010-01).

### Operation method

All operations were performed by the same surgeon (R.C.) under general anesthesia (propofol) with endotracheal intubation and muscle relaxants in the same endoscopy center. Carbon dioxide (CO_2_) was used for intraoperative endoscopic procedures. Patients were routinely placed in the left lateral position, and intravenous antibiotics were used prophylactically when deemed necessary based on preoperative comprehensive judgment. The surgical procedures used in this study were as follows.

#### Preoperative preparation

Routine fasting, blood routine, and coagulation tests were performed. Scopolamine butylbromide was used for spasmolysis if necessary.

#### Equipment and accessories

**Figure 1.**
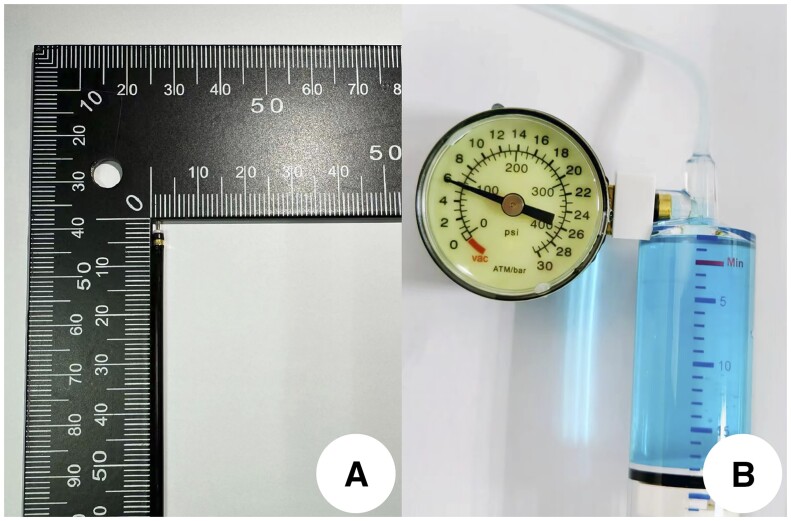
Knife head diameter and water pump pressure

The equipment and accessories for the operation were as follows: single-channel electronic colonoscope (PCF-Q260JI, Olympus Corporation, Japan), single-channel electronic gastroscope (GIF-Q260J, Olympus Corporation, Japan), transparent cover (D-201-11802, Olympus Corporation, Japan), disposable high-frequency cutting knife (Figure 1A; MK-T-2-195, Nanwei Medical Technology Co., Ltd., China), disposable endoscopic injection needle [25G (0.4 mm) × 230 mm, Nanwei Medical Technology Co., Ltd.], CO2 air pump (Olympus Corporation; Japan), ERBE electrocoagulation device with high-frequency generator (VIO200D, Elbo GMBH; Germany) and argon plasma coagulation device (APC300, Elbo GMBH; Germany), hemostatic forceps (CF195-AT, Nanwei Medical Technology Co., Ltd., China). In addition, 0.9% normal saline 250 mL with 0.4% indigo carmine 2 mL was used for submucosal injection, and the water-jet pressure for disposable balloon expandable pressure pump was 6 ATM/bar (Figure 1B).

#### Operation

Endo Cut Q, Effect 3, Duration 2, Interval 4, and Forced Coag 50w were used in all electrosurgical operation modes. If hemostatic forceps were required, they should be adjusted to Endo Cut Q, Effect 3, Duration 2, Interval 4, Soft Coag 80w. First, the location, size, and shape of the lesion were determined by detailed observation under therapeutic endoscopy. Then, appropriate endoscopic techniques were used to remove the lesions based on their characteristics. The general steps for all lesion resection methods are as follows: (i) after injecting the lesion submucosally with an injection needle, the lesion is well lifted; (ii) circumferentially incise the lesion; (iii) replenish the submucosal water pad through with “water injection knife”; (iv) gradually peel off the lesions completely.

**Figure 2.**
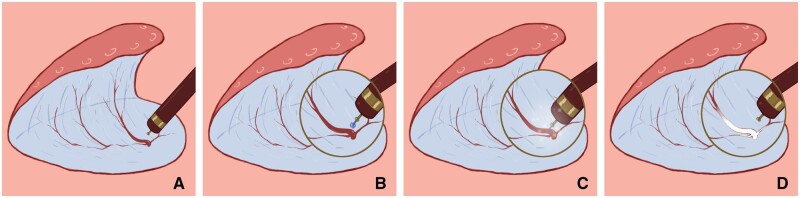
Intraoperative hemostasis with pretreatment of free vessels. (A) Submucosal vessels were exposed during lesion dissection. (B) After injecting the submucosa of the lesion using an injection needle, dissociate and uncover all accessible blood vessels during lesion dissection. Moreover, compare the blood vessels to the knife head to determine the thickness of the blood vessels. (C) The knife tip was placed close to the blood vessel, approximately 1 mm apart, and strengthens coagulation and hemostasis while injecting water. (D) Gradual “whity” of the vessels was seen until complete ischemic necrosis, after which the vessels were cut off.

**Figure 3.**
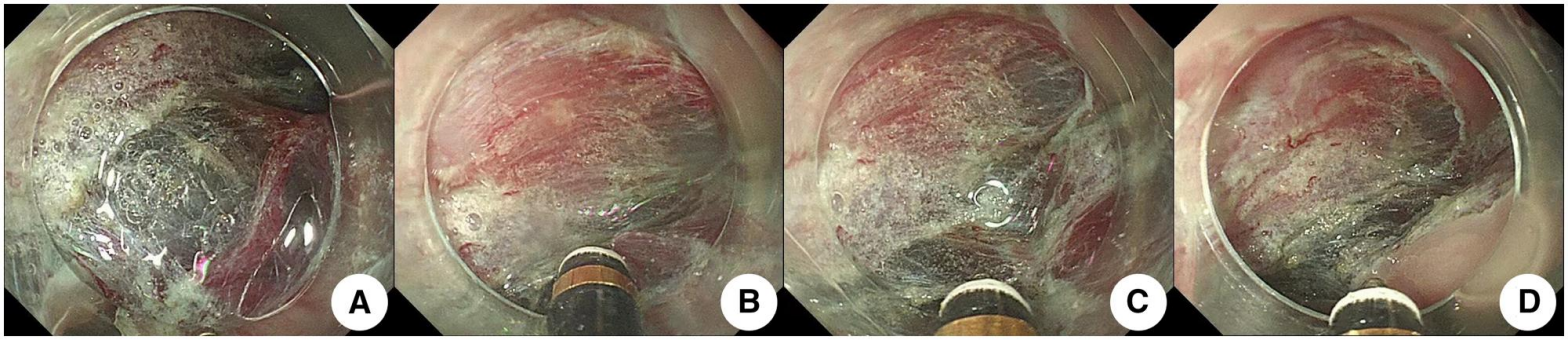
Intraoperative pretreatment of free blood vessels to stop bleeding. (A) Submucosal blood vessels were exposed when the lesion was dissected. (B) Submucosal water was injected, and the blood vessels were stripped off. The diameter of the blood vessels was measured by comparing them with the knife head. (C) The knife head was positioned close to the blood vessels, approximately 1 mm apart, and water was injected into the blood vessels, strengthening coagulation and stopping blood. (D) The vessels exhibited a gradual and complete “whity” state, and the vessel was cut off.

**Figure 4.**
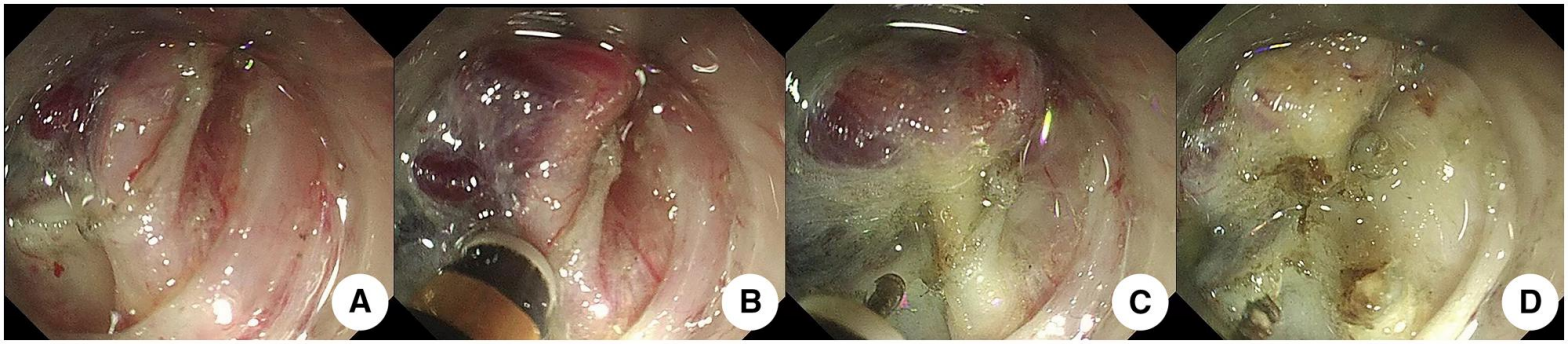
Intraoperative hemostasis with pretreatment of free blood vessels. (A) During the dissection of the lesion, the submucosal artery was exposed. (B) The knife was placed close to the vessel and electrocoagulated while injecting water. (C) Gradual “whity” of the artery was seen until complete ischemic necrosis. (D) The vessel was cut.

**Figure 5.**
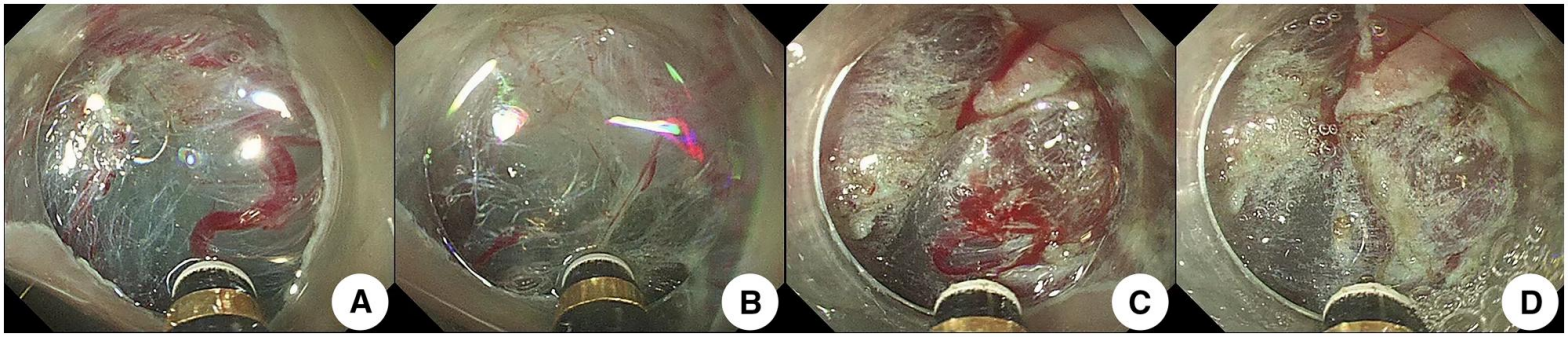
Intraoperative hemostasis with pretreatment of free blood vessels. (A) During the dissection of the lesion, the submucosal artery was exposed. (B) After performing submucosal water injection, continuously inject water while coagulating strongly for hemostasis. As the blood vessels gradually turn completely whitish, cut off the blood vessels. (C) During dissection, blood oozed from the vascular plexus, and the blood vessels were treated with electrocoagulation while water was being injected. (D) Vascular complete “whity” state, without an eschar.

If vascular or active bleeding occurs during surgery, the hemostasis steps are as follows:

*Pretreatment of free blood vessels for hemostasis during operation* (Figures 2–5): (i) the submucosal blood vessels were exposed, and all accessible blood vessels were dissociated and bare during lesion dissection; (ii) the diameter of the blood vessel was determined, taking the gold knife head as the reference, and the diameter of the knife head was about 0.8 mm (Figure 1A). The blood vessel was compared with the diameter of the knife head, and the blood vessel was divided into four grades of <1 mm, 1 mm, 2 mm, and 3 mm; (iii) the knife tip is in close proximity to the blood vessel, approximately 1 mm apart, and strengthen coagulation and hemostasis while injecting water; mediated by “water,” a slow heat conduction effect is produced; (iv) gradual “whity” of the vessels was seen until complete ischemic necrosis, after which the vessels were cut off. During the process of hemostasis, the degree of ischemic necrosis of blood vessels is evaluated based on the degree of “whitening” of the transverse diameter of the blood vessels; as shown in Figures 3–5, the blood vessels gradually turn from red (veins) or pink-white (arteries) to completely white. After it was determined that the blood vessel had reached complete ischemic necrosis, it was transected. The degree of vessel whitening was graded according to the following criteria: (i) whitening of one-third of the transverse diameter was defined as 1 point (Figure 3B); (ii) whitening of two-thirds was defined as 2 points (Figure 3C); (iii) complete whitening was defined as 3 points (Figure 3D). After reaching 3 points, the blood vessel was cut off and observed for 10–30 seconds to ensure that no active bleeding was observed. When the broken blood vessel stump has solidified, hemostasis is complete.

**Figure 6.**
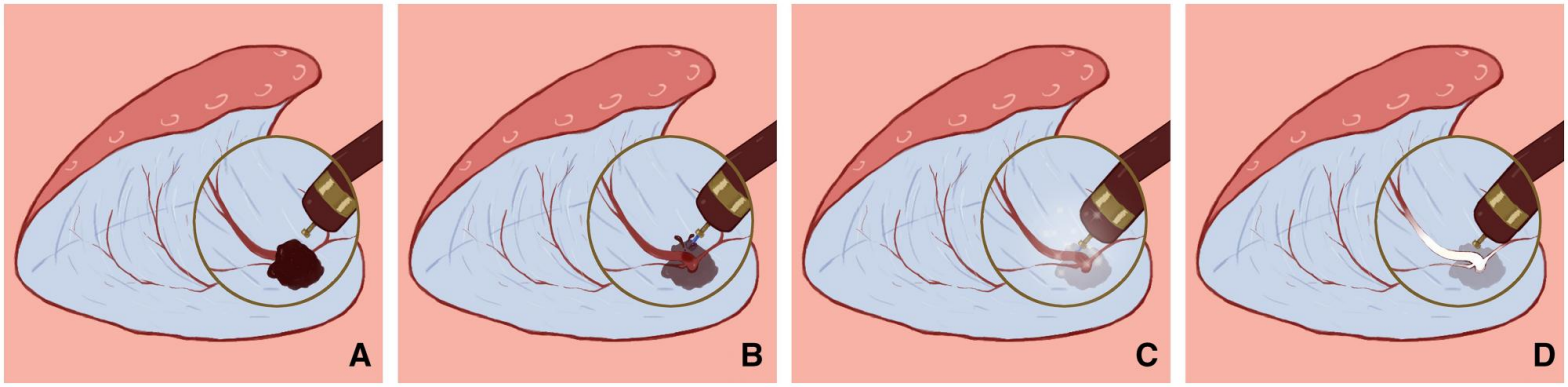
Intraoperative hemostasis of active bleeding. (A) Active bleeding. (B) The water injection port of the cutting knife is injected into the bleeding point, and the blood is cleaned up. (C) The bleeding point is exposed, and the submucosa is raised by water injection at the edge, and the edge is strongly coagulated. (D) The blood vessel is “whity” and the bleeding point is stopped.

**Figure 7.**
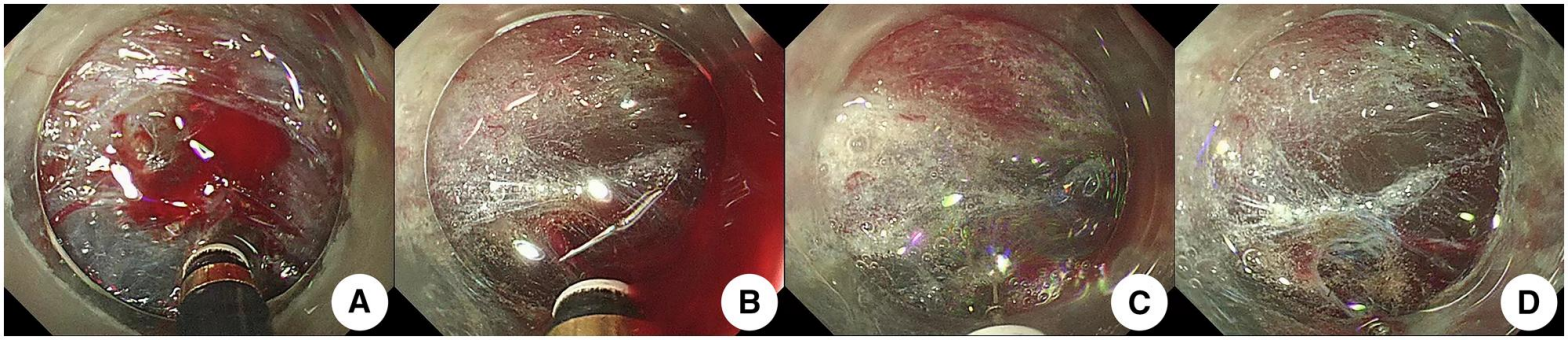
Intraoperative hemostasis of active bleeding. (A) Active bleeding. (B) The water injection port of the cutting knife was inserted into the bleeding point and effectively cleaned it. (C) The bleeding point was exposed, and the submucosa was raised by injecting water at the edge, and the edge was strongly coagulated and stopped bleeding. (D) Vascular complete “whity” state, without an eschar.

*Intraoperative active bleeding hemostasis method* (Figures 6 and 7): (i) the water injection port of the cutting knife was injected with water jet against the bleeding site to clean the blood and expose the bleeding site; (ii) the submucosa was elevated by injecting water with the cutting knife, and strong coagulation was performed at the same time; (iii) electrocoagulation was stopped after the bleeding stopped at the bleeding site. All postoperative wounds were treated with “water-jet” electrocoagulation.

The criteria for conversion to the use of hemostatic forceps are as follows: (i) vessels or vascular plexus larger than 3 mm in diameter; (ii) complete hemostasis cannot be achieved by the “water-jet” hemostasis method within 30 seconds; (iii) when the operator judges that there would be difficulty for “water-jet” hemostasis, hemostatic forceps should be used directly for the safety of the patient; (iv) blood vessels that are not fully exposed because it is difficult to use “water-jet” to stop bleeding. If any of the above points are met, hemostatic forceps are used to stop bleeding. The lesion was removed, and all postoperative wounds were treated by “water-jet” electrocoagulation.

#### Postoperative treatment

Patients with intragastric lesions were fasted after surgery, and a gastrointestinal decompression tube was inserted. Symptoms such as fever, abdominal pain, abdominal distension, and peritoneal irritation, as well as the color and volume of the gastric tube drainage were observed. Proton pump inhibitors were intravenously infused simultaneously. The decision to administer perioperative antibiotics was based on the individual patient’s condition. If the patient’s condition remained stable, the gastric tube was removed 48 hours after surgery, and a fluid diet was administered. If there was no discomfort after the fluid diet, the patient could be discharged for observation. After being discharged, patients continued on a liquid diet for 1 week, followed by a semi-liquid diet for another week. They were also prescribed oral proton pump inhibitors for 8 weeks.

Those with intestinal lesions were fasted after surgery and observed for fever, abdominal pain, abdominal distension, peritoneal irritation, and color of stool. The decision to use antibiotics was based on the patient’s perioperative condition. If the patient’s condition remained stable during the 48-hour observation period, fluid diet was administered. If there is no discomfort after the fluid diet, the patient can be discharged for observation. After discharge, the fluid diet was continued for 1 week and then transitioned to a semi-liquid diet for another week.

### Data collection

Data were collected, including surgical data, adverse events, and length of hospital stay. Surgical data included lesion location, maximum diameter of lesion, maximum diameter of blood vessel, histopathology, and depth of the lesion. Adverse events included fever, bleeding, abdominal pain, perforation, delayed bleeding, and electrocoagulation syndrome. The primary outcomes of this study are the incidence rates of adverse events and R0 resection, and the secondary outcomes are length of hospital stay and short- and long-term outcomes.

### Follow-up

Patients were followed up by telephone for 1 month after discharge from the hospital and asked if they had any complaints such as hematochezia, abdominal pain, fever, etc. for short-term outcomes.

Patients also underwent regular endoscopy, tumor marker testing, and imaging examinations at 3, 6, and 12 months after gastric ESD for long-term outcomes. Those with no residue or recurrence were followed up once a year with the same items as before, while patients with residue or recurrence were followed up every 3 months and once a year after complete removal of the lesions.

For patients with complete R0 resection in colorectal ESD, we recommend re-examination of colonoscopy within 1 year. If the horizontal margin of the tumor is difficult to evaluate, colonoscopy is recommended within 6–12 months [4, 5].

### Statistical analysis

Data are presented as mean ± standard deviation or median and interquartile range (IQR) according to the data distribution.

## Results

A total of 10 patients with gastric or intestinal mucosal lesions were identified by gastroscopy or colonoscopy due to related symptoms and met the absolute indication for endoscopic treatment. The size of the surgical wounds ranged from approximately 2–8 cm. All the tumors that met the absolute indications for endoscopic treatment were successfully removed by ESD in a single procedure. The “water-jet” hemostasis method was used to stop bleeding during the operation.

Data on patients treated with endoscopic hemostasis using the “water-jet” method are shown in Table 1. The median age of the patients was 52.00 (IQR, 50.00–52.75) years old, and the proportion of male patients was 70%. Among them, there were five lesions in the stomach and five in the intestine. The median maximum lesion diameter was 4.00 (3.25–4.75), and the median maximum vessel diameter was 3 (2–3) mm. Pathologic results showed R0 resection in all samples. The median length of hospitalization was 4 (3–4.75) days. In case 5, the “water-jet” hemostasis failed to stop bleeding in a blood vessel, and then thermocoagulation hemostasis with hemostatic forceps was switched during the process of stripping, and the “water-jet” hemostasis method was used in the subsequent stripping process. There was no intraoperative perforation. There were no adverse complications, such as delayed bleeding or electrocoagulation syndrome, after surgery.

**Table 1.** Data of the 10 patients treated with endoscopic hemostasis by “water-jet” method

No.	Sex	Age, years	Lesion location	Maximum diameter of lesion, cm	Maximum diameter of the blood vessel, mm	Histopathology	Change to hemostatic forceps to stop bleeding	Depth of the lesion	R0 resection	Length of hospital stay, days	Adverse event	Endoscopic result at 6 months after surgery
1	Male	57	Distal portion of the gastric body	4	2	Highly differentiated adenocarcinoma	No	Muscularis mucosae	Yes	3	No	No recurrence and residue
2	Male	46	Middle part of gastric body	5	3	Moderately differentiated tubular adenocarcinoma	No	Submucosa 90 µm	Yes	4	No	No recurrence and residue
3	Male	52	Under the cardia	4	3	Highly differentiated adenocarcinoma	No	Muscularis mucosae	Yes	4	No	No recurrence and residue
4	Male	45	Rectum	3	2	Traditional serrated adenoma	No	Mucosae	Yes	3	No	No recurrence and residue
5	Female	50	Rectum	3	2	Tubulovillous adenoma	Yes	Mucosae	Yes	3	No	No recurrence and residue
6	Female	50	Transverse colon	3	3	Tubulovillous adenoma	No	Mucosae	Yes	4	No	No recurrence and residue
7	Male	53	Ascending colon	5	3	Tubulovillous adenoma	No	Mucosae	Yes	5	No	No recurrence and residue
8	Male	52	The lesser curvature of the antrum	4	3	Moderately differentiated tubular adenocarcinoma	No	Lamina propria mucosae	Yes	3	No	No recurrence and residue
9	Male	55	Sigmoid colon	4	2	Tubular adenoma	No	Mucosae	Yes	5	No	No recurrence and residue
10	Female	52	Fundus of stomach	5	3	Intestinal adenoma	No	Mucosae	Yes	5	No	No recurrence and residue

R0 = en bloc resection with negative lateral and vertical margins.

## Discussion

The endoscopic technique of ESD, which is minimally invasive, efficient, and economical, has been widely used in the treatment of early gastrointestinal cancers and precancerous lesions. Preventing and controlling bleeding related to ESD has become a challenging problem for medical staff [6, 7]. How to quickly and accurately evaluate the risk of ESD bleeding while mastering ESD operation skills to avoid the occurrence of bleeding during and after ESD is a necessary condition to ensure the safety of ESD surgery. Adequate hemostasis planning for patients with high-risk factors for ESD bleeding can effectively reduce the incidence of ESD bleeding. During the operation, the surgeon’s surgical skills, hemostasis methods, and vessel pretreatment are crucial in preventing complications. Intraoperative pretreatment of blood vessels and hemostasis at the bleeding site can significantly reduce the incidence of intraoperative bleeding [7]. The methods of intraoperative endoscopic hemostasis include drug-induced hemostasis and physical hemostasis. Drug-induced hemostasis mainly includes spraying norepinephrine, using hemostatic powder, and applying thrombin [8, 9]. Physical hemostasis mainly includes argon plasma coagulation, electrocoagulation (suitable for vessels ≤2 mm in diameter), thermal tissue clamp (suitable for vessels >2 mm in diameter), and the use of a hemostatic clip, over-the-scope clip (OTSC), etc. Although the aforementioned hemostatic measures have been validated in clinical practice, they also possess certain limitations, such as the slow onset of drug hemostasis and uncertainty of the hemostatic effect. Repeated electrocoagulation hemostasis can easily result in excessive tissue carbonization, leading to inadequate lifting of the submucosal injection, unclear submucosal layers, and affecting lesion dissection. Excessive electrocoagulation can cause tissue damage and may even result in intraoperative gastrointestinal perforation, delayed perforation, and electrocoagulation syndrome. In the case of active bleeding, the reaction between electrocoagulation and hemoglobin can easily produce eschar, which not only affects the surgical field and the submucosal water pad supplement, resulting in difficult dissection but also easily covers the tip of the knife, affecting the effectiveness of electrocoagulation and electroresection. Hemostasis of the lesion vessels and surrounding tissues using hemostatic forceps can reduce the height of the submucosal uplift, which may lead to an increased risk of perforation. The clips can affect the operator’s field of vision and impede other parts of bleeding. Therefore, it is very important to use reasonable endoscopic hemostasis during the operation.

With the continuous development of endoscopic equipment, water-jet equipment has been continuously improved, including the gradual rise of instruments with water injection equipment, such as “water-jet” knife. Repici *et al.* [10] mentioned that submucosal injection by using water injection knife instead of injection needle can not only reduce the time of replacement of accessories, but also maintain the submucosal water pad continuously, so as to improve the safety and effectiveness of resection treatment. In clinical practice, we found that the application of “water-jet” knife in ESD not only has the above advantages, but can also be skillfully used to stop bleeding during operation. In this method, saline is used in both “water-jet” and submucosal injection because it can provide better conductivity for effective blood coagulation. Salt water contains dissolved electrolytes such as sodium and chloride ions, which increase its electrical conductivity and ability to transmit the current needed to clot and stop bleeding during electrosurgery [11]. In general, the “water-jet” method of hemostasis has the following advantages: (i) When the blood vessel is strongly coagulated, the heat of the knife can be taken away. When the blood vessel is stopped, it is gradually approached from the edge of the blood vessel, playing the effect of “boiling the frog in warm water,” and the blood vessel is slowly similar to the effect of “soft coagulation.” It is also applicable for vessels larger than 2 mm in diameter. (ii) Because of the mild effect, the blood vessel is not immediately cut, so the broken end of the blood vessel is not fully electrocoagulated and occluded, resulting in massive bleeding. (iii) For active bleeding, the high-pressure water injection function of the knife head can be used to help clean up the blood and quickly locate the bleeding point. (iv) For bleeding vessels at the broken end, clean up the blood while injecting water, take away the blood, and avoid the formation of eschar during hemostasis. (v) During water injection, the submucosal water pad can be fully supplemented, which can raise the submucosal elevation and prevent perforation during hemostasis. (vi) It can be used for large vessels, such as those greater than 2 mm, to reduce the time spent on replacing instruments (such as thermal tissue forceps). (vii) When the edge of the lesion was dissected, the submucosal blood vessels were found, and the “water injection” hemostasis method could ensure a sufficient submucosal water cushion and increase the height of blood vessel elevation. It avoids the unclear stratification of mucosa and muscle layer caused by insufficient water storage at the lesion edge during electrocoagulation trimming and cutting, and the adhesion between mucosa and muscle layer is easy to occur during electrocoagulation and hemostasis, resulting in the difficulty of making mucosal flap. (viii) Taking away heat from the cutter head while injecting water can reduce the generation of smoke during the cutting process and keep the surgical field clear. (ix) This method is simple to operate, easy to learn, and easy for the operating physician to master.

**Figure 8.**
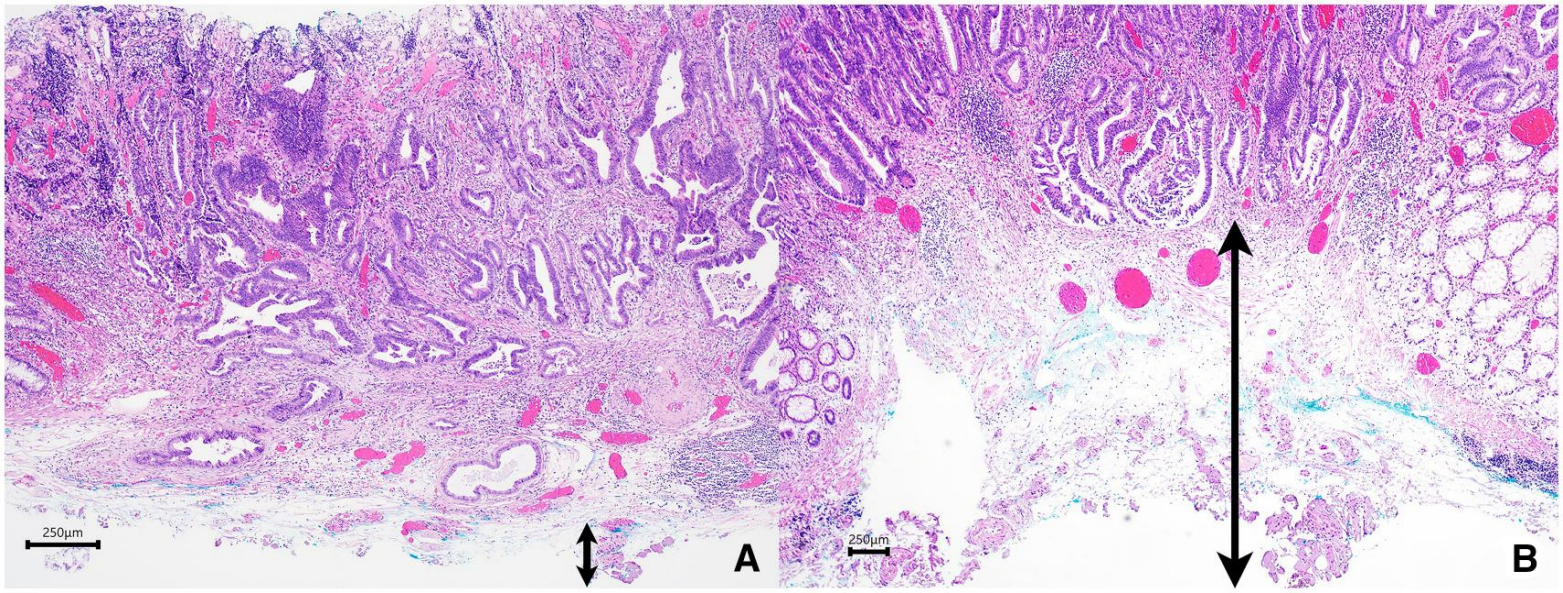
Effect of high-frequency current electrification on endoscopic resection specimens. (A) Tissue basement damage caused by “water injection” hemostasis. (B) Tissue basement damage caused by thermocoagulation forceps hemostasis.

In the 10 cases we included, extra electrocoagulation forceps was used for a naked vessel due to insufficient “water-jet” hemostasis, and the diameter of the vessel was about 2 mm. We considered that the vascular “whitening” was not complete because it did not keep a certain distance from the blood vessel or was not fully injected during the operation, which directly cut off the incomplete ischemic necrotic blood vessels, resulting in bleeding, and the remedial measures taken were thermocoagulation forceps to stop bleeding. In clinical ESD practice, for vessels larger than 2 mm in diameter, it is difficult to use the tip of the incision knife alone to achieve the purpose of pretreatment and hemostasis, and it is often necessary to replace the thermocoagulation forceps. The “water-jet” hemostasis method is adopted; through the slow thermocoagulation effect, the blood flow in the blood vessel is slowly solidified, and after blocking the blood flow, the vessel is cut (Figure 4), so as to achieve reliable preventive hemostasis. After practice, for intraoperative active bleeding from vessels >2 mm, water injection hemostasis is generally difficult to stop bleeding successfully, and we will take thermocoagulation forceps to stop bleeding. So far, we have tried to use only water injection knife to stop bleeding by “water-jet,” and the maximum diameter of vascular plexus for hemostasis is 3 mm. We compared the same ESD specimens with thermocoagulation forceps after the failure of “water-jet” hemostasis, and we can see the effect of high-frequency current on endoscopic resection specimens. Because the tissue is heated by high-frequency current, the protein components of the tissue cells are denatured by thermal coagulation, which also causes damage to the tissue of the cutting edge of the base. In contrast, the degree of injury of “water-jet” hemostasis is lower than that of thermocoagulation forceps (Figure 8). It is suggested that the thermocoagulation damage of the specimen by “water-jet” hemostasis method is relatively small, thus reducing the influence of pathologists on the judgment of lesion depth.

There are some limitations to this study. First, the number of cases included was small, and the sample size needs to be further increased to verify the conclusion of the study. Second, this is a prospective, single-arm, and single-center study. In the future, multi-center, randomized controlled, and high-quality clinical research is needed to further explore the application of “water-jet” hemostasis in gastrointestinal ESD. Third, intraoperative bleeding, perforation, and delayed bleeding were also related to objective factors such as surgeon technique and lesion location. Fourth, the golden knife was used in all cases within this group, and the parameters of different water-jet knives vary, making standardization difficult and requiring further exploration. The water-injection knife was used in all cases, and the electrosurgical operation mode used Endo Cut Q, Effect 3, Duration 2, Interval 4, and Forced Coag 50w. The water-jet pressure was 6 ATM/bar. Different water injection knives have different parameters and are difficult to standardize. It is necessary to further explore the optimal water-jet pressure and electrocautery settings for other types of knives to provide standardized settings for clinical use. Fifth, the price of water-injection knife is generally higher than that of non-water-injection knife, and the cost of surgery may be relatively high.

## Conclusions

In general, the “water-jet” hemostasis method in ESD is effective, safe, and feasible. It is a kind of water-jet knife, while filling water, taking away the heat of the knife head, playing a “warm water boiling frog” effect, thus forming a similar “soft coagulation” effect. This technique deserves widespread promotion. We hope that with more cases in the future, we will be able to bring good news to more patients.

## Supplementary Material

goae088_Supplementary_Data
